# PS-02 - OUTBREAK MANAGEMENT AND INCIDENT RESPONSE: PUBLIC HEALTH INVESTIGATION OF BOTULISM CASES ASSOCIATED WITH UNLICENSED COSMETIC BOTULINUM TOXIN USE

**DOI:** 10.1093/pubmed/fdag046.051

**Published:** 2026-07-09

**Authors:** Mrs Samantha Charlesworth, Mrs Sara Coy, Dr James McGowan

**Affiliations:** UK Health Security Agency; UK Health Security Agency; UK Health Security Agency

## Abstract

**Background:**

Botulism is a rare but potentially life-threatening condition requiring rapid identification and coordinated public health action. In 2025, an unexpected increase in suspected botulism cases was identified in the East of England, prompting a regional and national health protection response. Botulinum toxin may only be supplied and administered for cosmetic purposes under a valid prescription issued by an appropriate healthcare professional.

**Objectives:**

To describe the outbreak management and incident response to a cluster of suspected botulism cases and to identify key public health learning relevant to prevention, regulation, and multi-agency working.

**Methods:**

An incident was declared following notification of suspected cases presenting with neurological symptoms consistent with botulism. The East of England health protection team led a regional investigation alongside a national enhanced incident response, with cross-regional collaboration. Enhanced surveillance, active case finding, and structured exposure histories were undertaken. Primary liaison was established with the local environmental health team and the Medicines and Healthcare products Regulatory Agency (MHRA), supported by NHS clinicians. Information from clinical and regulatory enquiries informed risk assessment and enforcement activity, including search of a premises and seizure of evidence.

**Results:**

The investigation identified a common exposure to cosmetic botulinum toxin injections administered by a single beauty practitioner using unlicensed products. Coordinated multi-agency and cross-regional action enabled risk mitigation, clinical follow-up, and prevention of further harm. Challenges included identifying informal cosmetic providers and navigating complex regulatory pathways.

**Conclusions:**

This incident highlights the importance of early pattern recognition, effective information sharing, and strong collaboration between health protection teams, environmental health, and national regulators. The learning is relevant across the 5 Nations and reinforces the need for improved oversight and awareness of medicines regulatory requirements within the non-medical cosmetic sector. The expanding non-medical cosmetic sector presents emerging risks where these regulatory requirements are not met.

